# Supplementary material for: A Novel Method for the Determination of Squalene, Cholesterol and Their Oxidation Products in Food of Animal Origin by GC-TOF/MS
Source: Int J Mol Sci. 2024 Feb 28;25(5):2807. doi: 10.3390/ijms25052807 (PMC10931826; doi:10.3390/ijms25052807)
Supplement: Supplementary file 1 [file ijms-25-02807-s001.zip › ijms-2871961-supplementary.pdf]

Article: Novel method for the determination of squalene, cholesterol and their oxidation products in food of animal origin by GC-TOF/MS

Supplementary materials

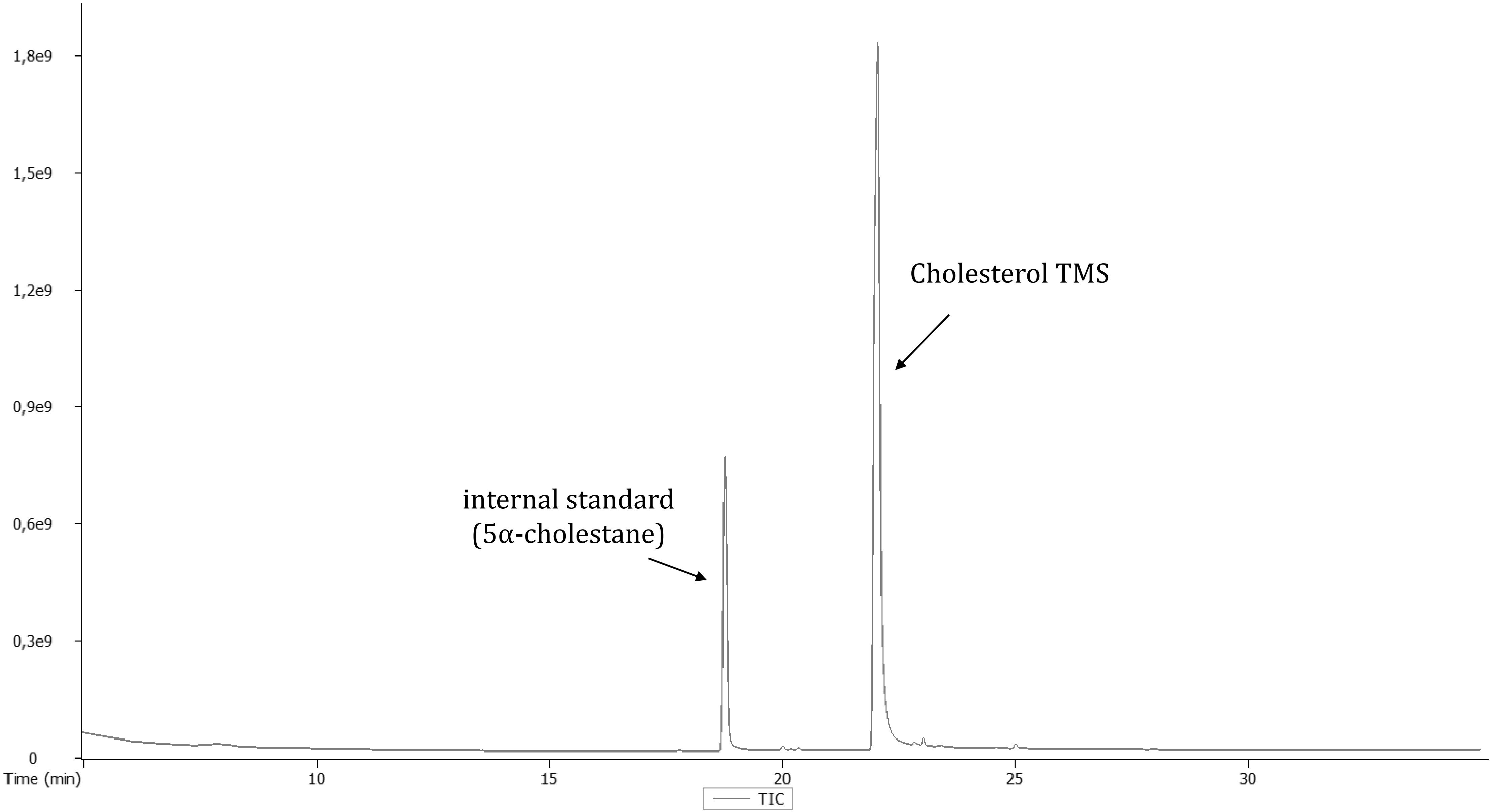

Figure S1. Total ion chromatogram of cheese sample.

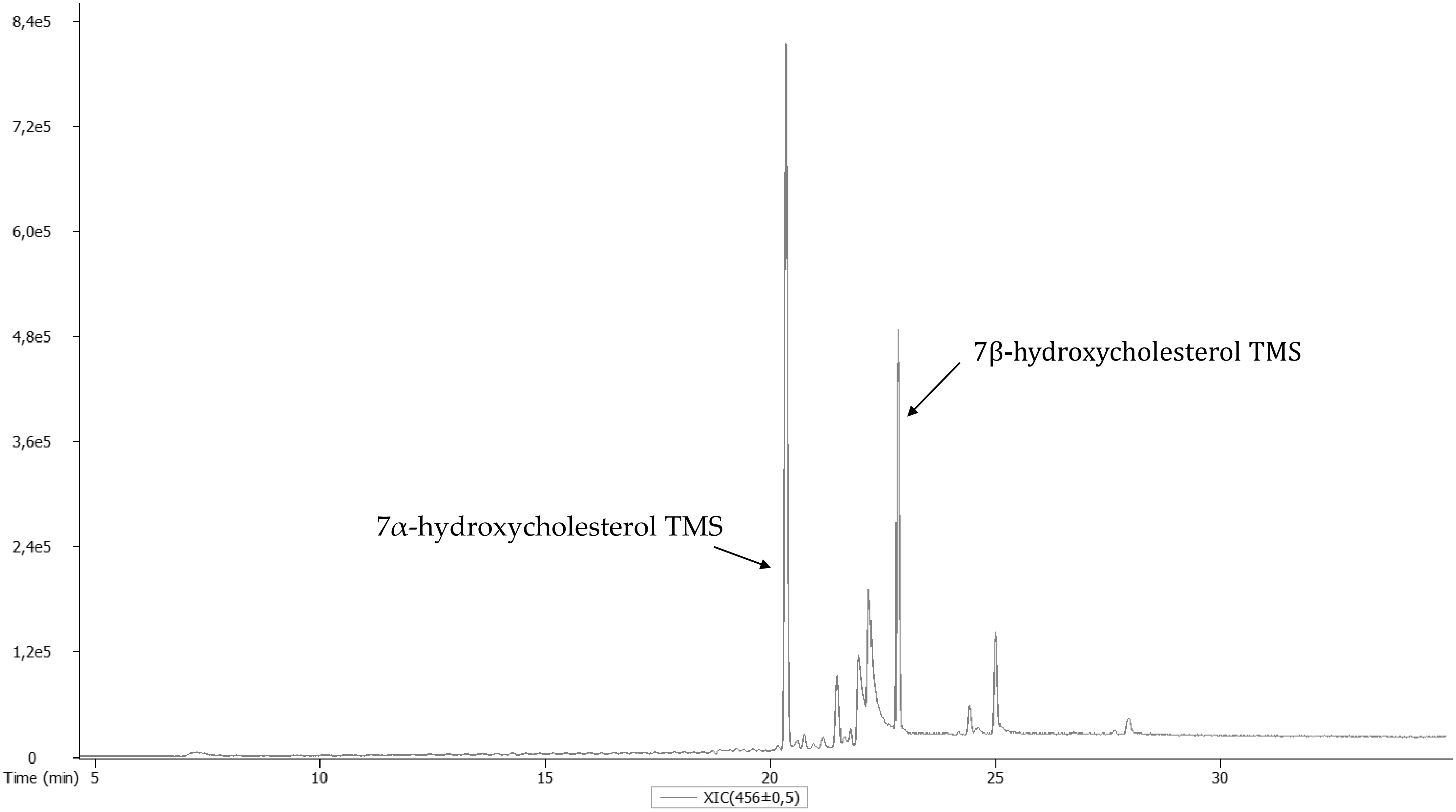

Figure S2. Extracted ion chromatogram (XIC, 456 Da) of cheese sample.

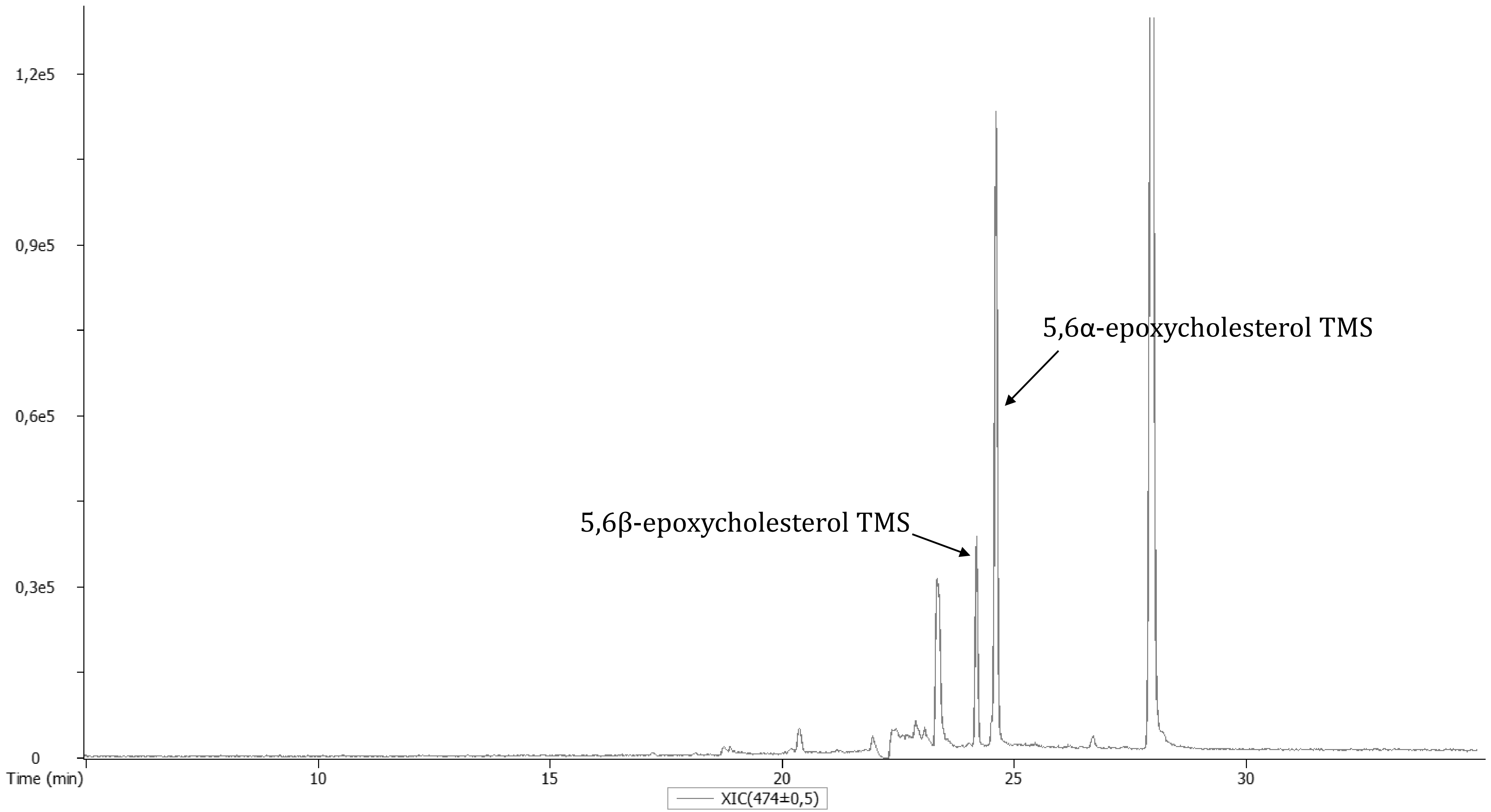

Figure S3. Extracted ion chromatogram (XIC, 474 Da) of cheese sample.

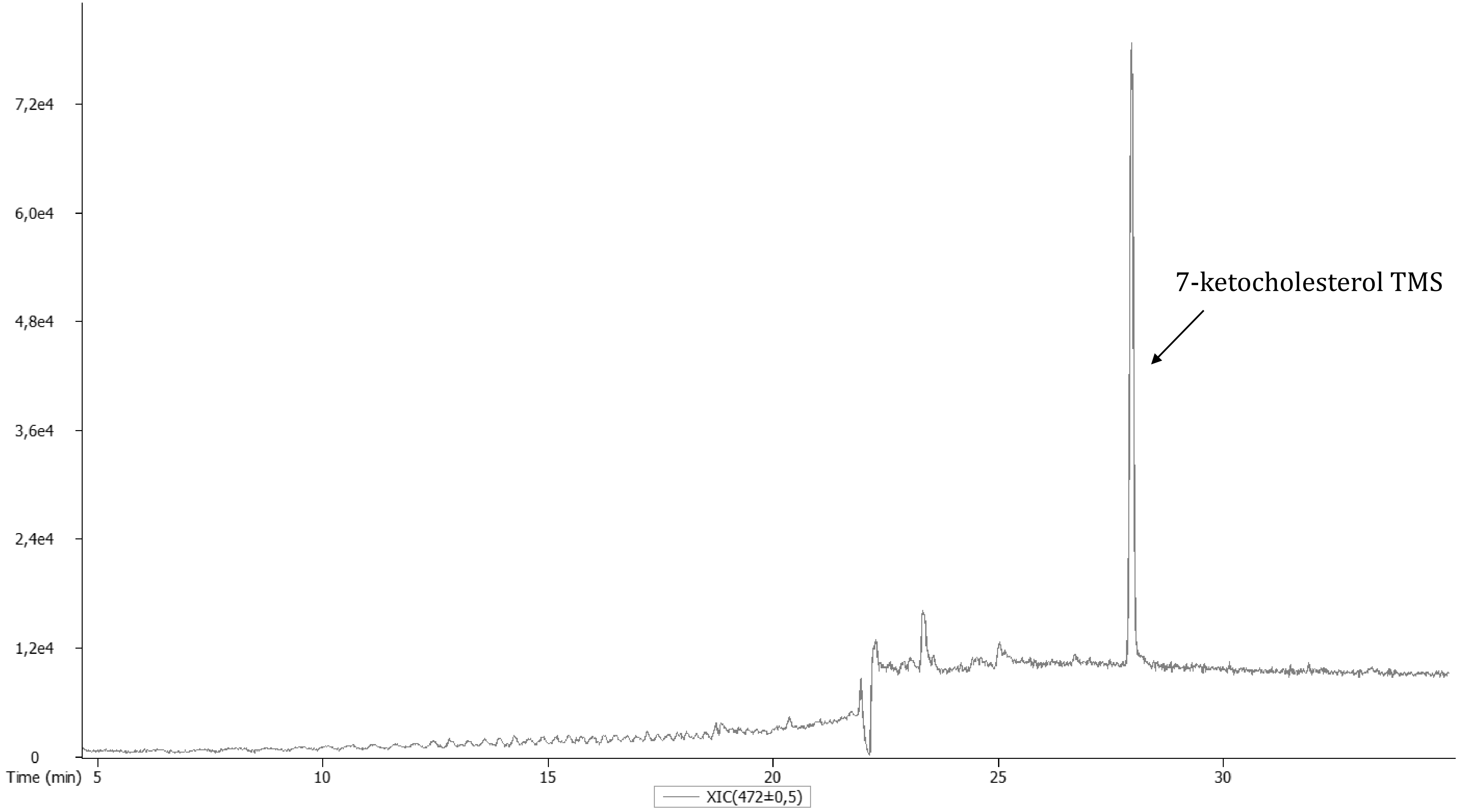

Figure S4. Extracted ion chromatogram (XIC, 474 Da) of cheese sample.

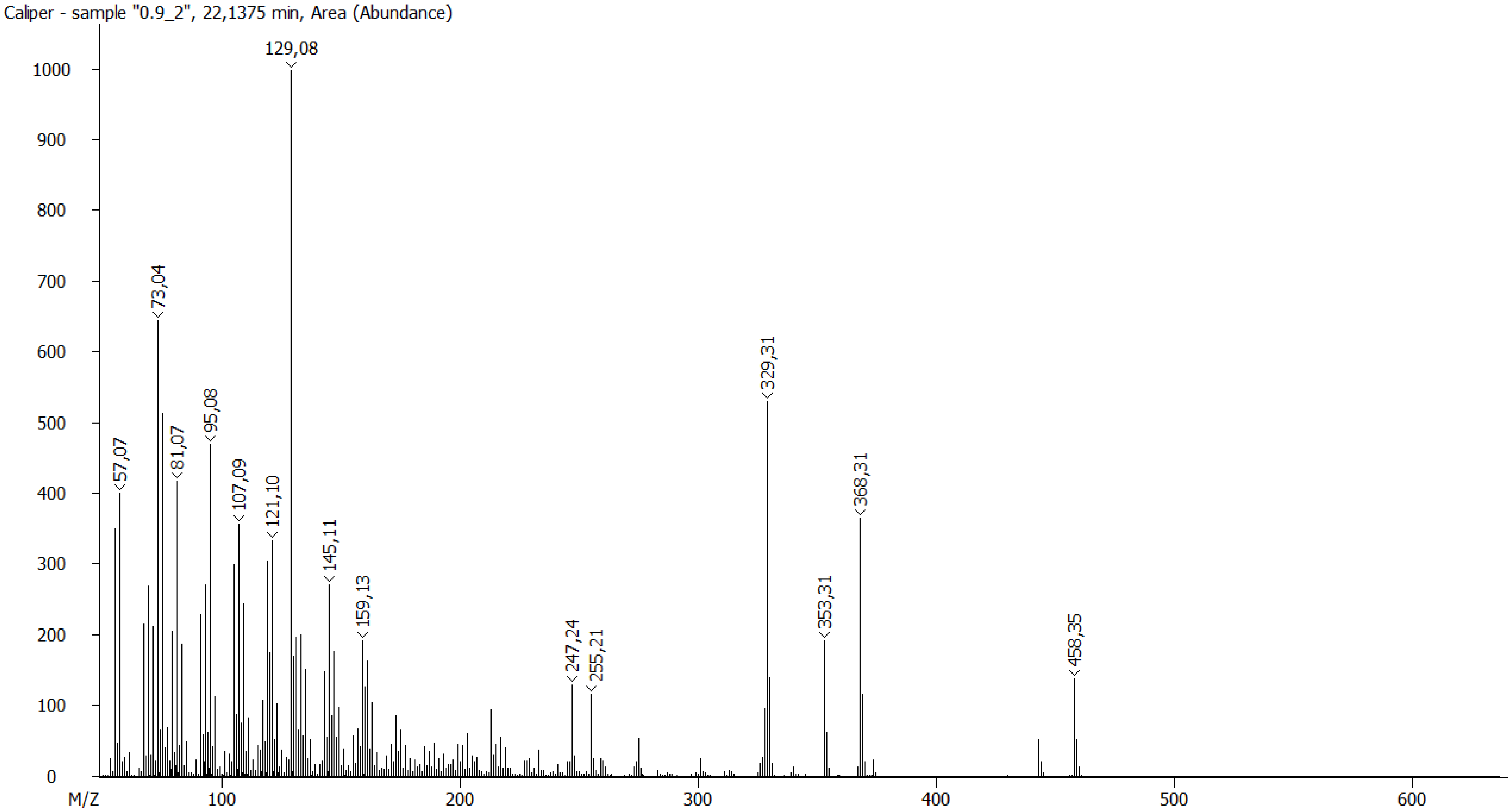

Figure S5. Mass spectrum of cholesterol TMS.

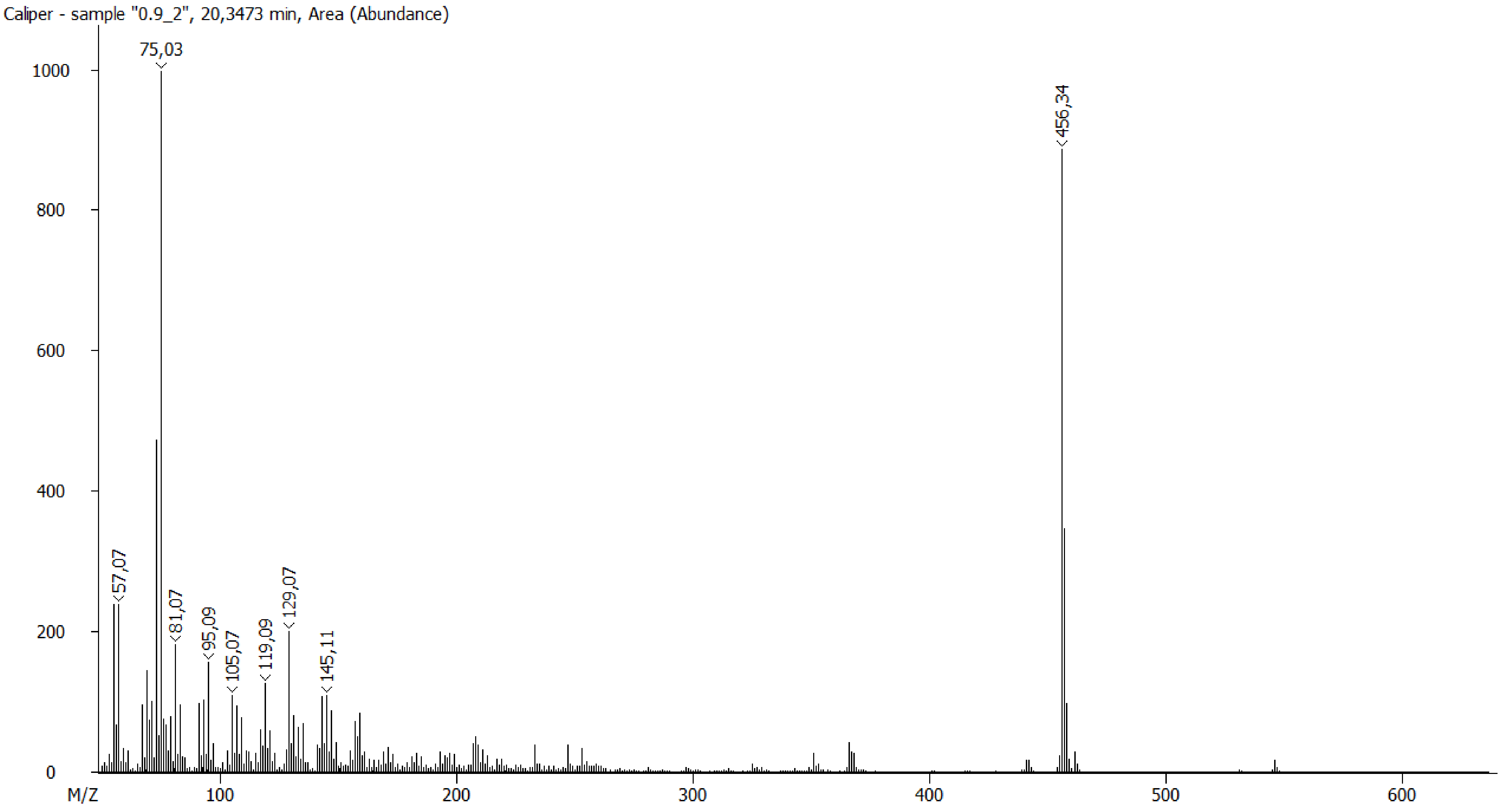

Figure S6. Mass spectrum of 7α-hydroxycholesterol TMS.

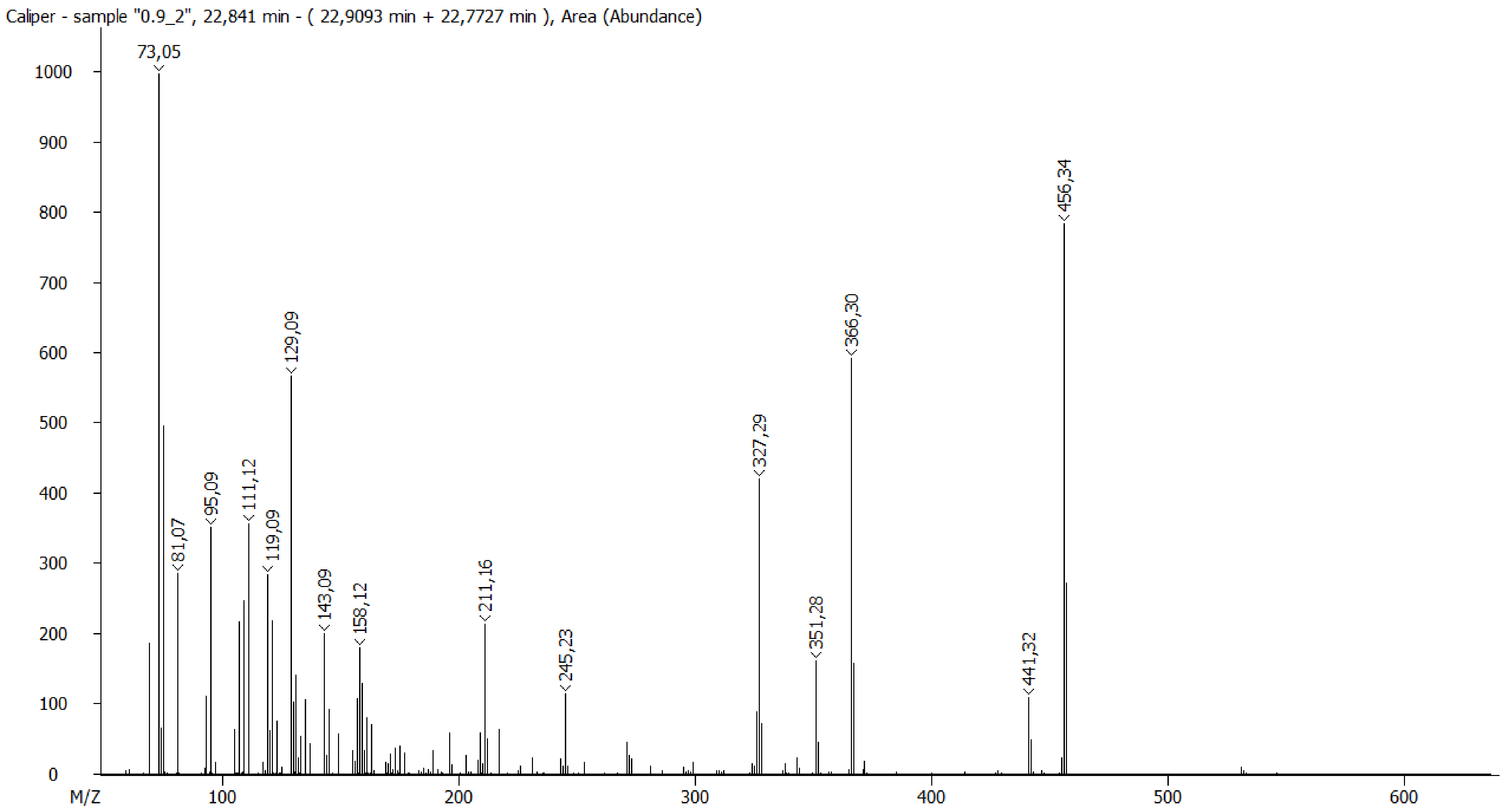

Figure S7. Mass spectrum of 7β-hydroxycholesterol TMS.

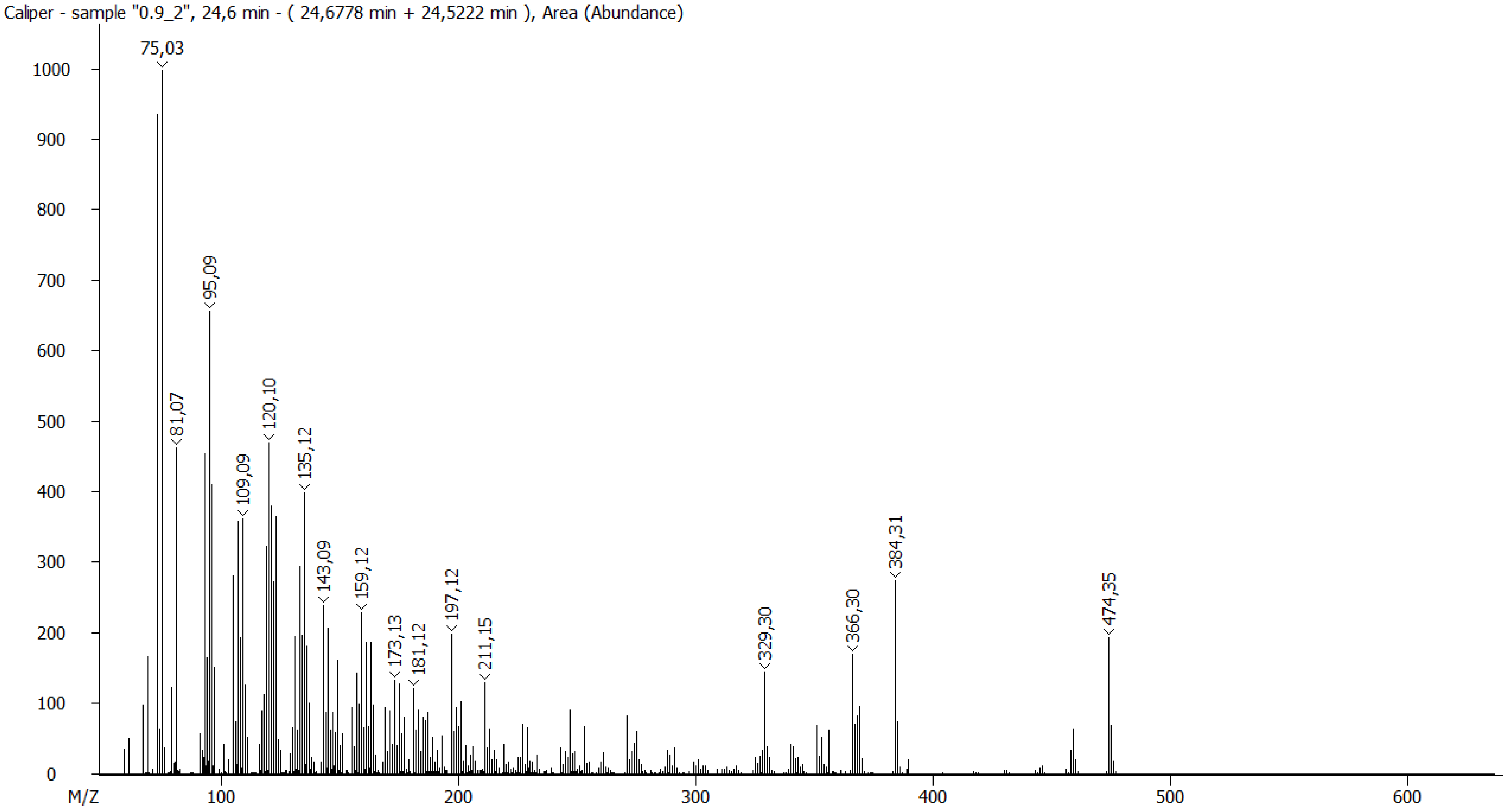

Figure S8. Mass spectrum of 5,6α-epoxycholesterol TMS.

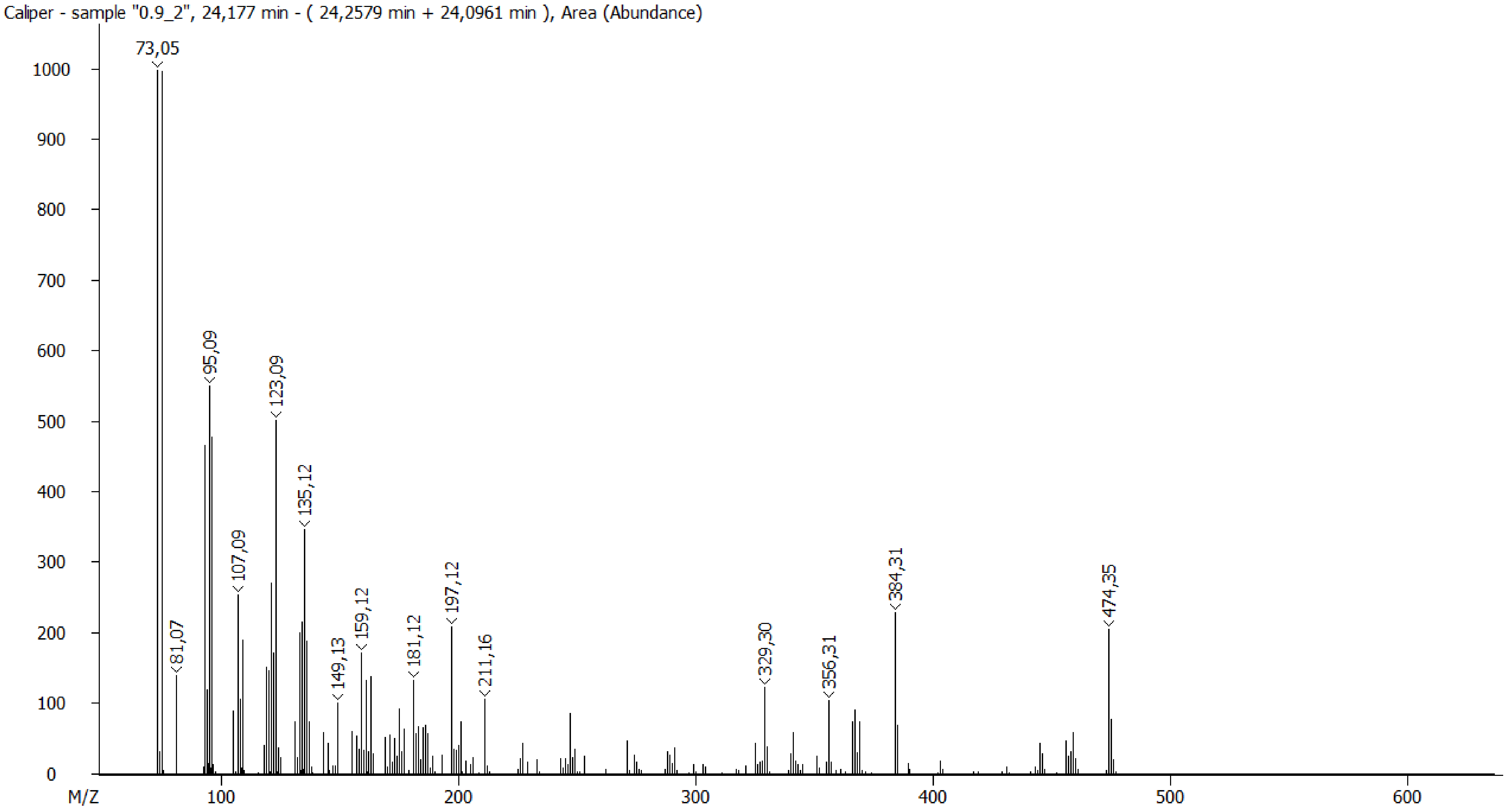

Figure S9. Mass spectrum of 5,6β-epoxycholesterol TMS.

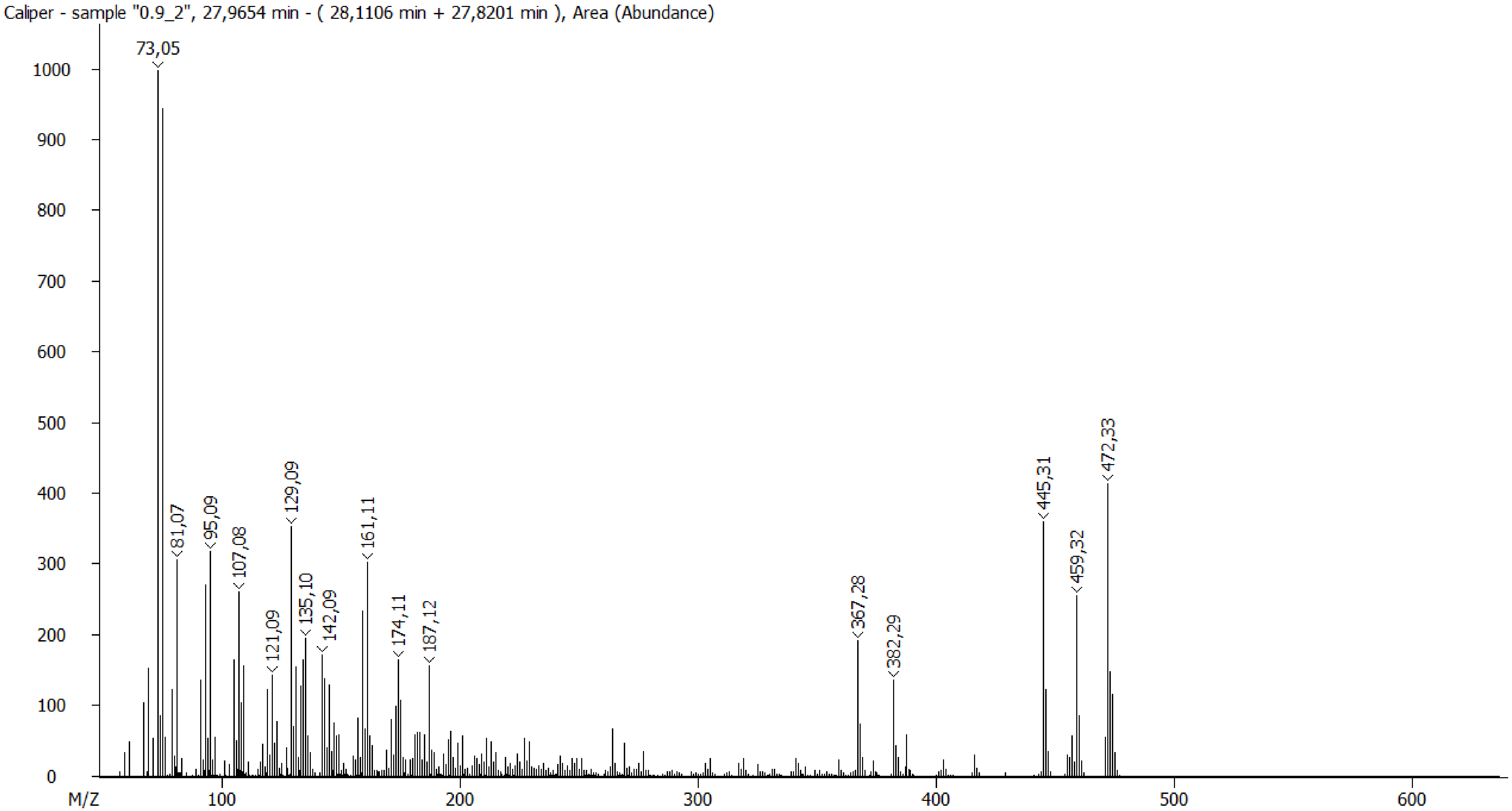

Figure S10. Mass spectrum of 7-ketocholesterol TMS.
